# Developing a pro-angiogenic placenta derived amniochorionic scaffold with two exposed basement membranes as substrates for cultivating endothelial cells

**DOI:** 10.1038/s41598-021-01922-y

**Published:** 2021-11-18

**Authors:** Siavash Shariatzadeh, Sepehr Shafiee, Ali Zafari, Tahereh Tayebi, Ghasem Yazdanpanah, Alireza Majd, Arvin Haj-Mirzaian, Soheyl Bahrami, Hassan Niknejad

**Affiliations:** 1 Department of Pharmacology, School of Medicine, Shahid Beheshti University of Medical Sciences, Tehran, Iran; 2 Department of Ophthalmology, Baylor College of Medicine, Houston, TX USA; 3 Ludwig Boltzmann Institute for Experimental and Clinical Traumatology in AUVA Research Center, Vienna, Austria

**Keywords:** Regeneration, Translational research

## Abstract

Decellularized and de-epithelialized placenta membranes have widely been used as scaffolds and grafts in tissue engineering and regenerative medicine. Exceptional pro-angiogenic and biomechanical properties and low immunogenicity have made the amniochorionic membrane a unique substrate which provides an enriched niche for cellular growth. Herein, an optimized combination of enzymatic solutions (based on streptokinase) with mechanical scrapping is used to remove the amniotic epithelium and chorion trophoblastic layer, which resulted in exposing the basement membranes of both sides without their separation and subsequent damages to the in-between spongy layer. Biomechanical and biodegradability properties, endothelial proliferation capacity, and in vivo pro-angiogenic capabilities of the substrate were also evaluated. Histological staining, immunohistochemistry (IHC) staining for collagen IV, and scanning electron microscope demonstrated that the underlying amniotic and chorionic basement membranes remained intact while the epithelial and trophoblastic layers were entirely removed without considerable damage to basement membranes. The biomechanical evaluation showed that the scaffold is suturable. Proliferation assay, real-time polymerase chain reaction for endothelial adhesion molecules, and IHC demonstrated that both side basement membranes could support the growth of endothelial cells without altering endothelial characteristics. The dorsal skinfold chamber animal model indicated that both side basement membranes could promote angiogenesis. This bi-sided substrate with two exposed surfaces for cultivating various cells would have potential applications in the skin, cardiac, vascularized composite allografts, and microvascular tissue engineering.

## Introduction

**Figure 1:**
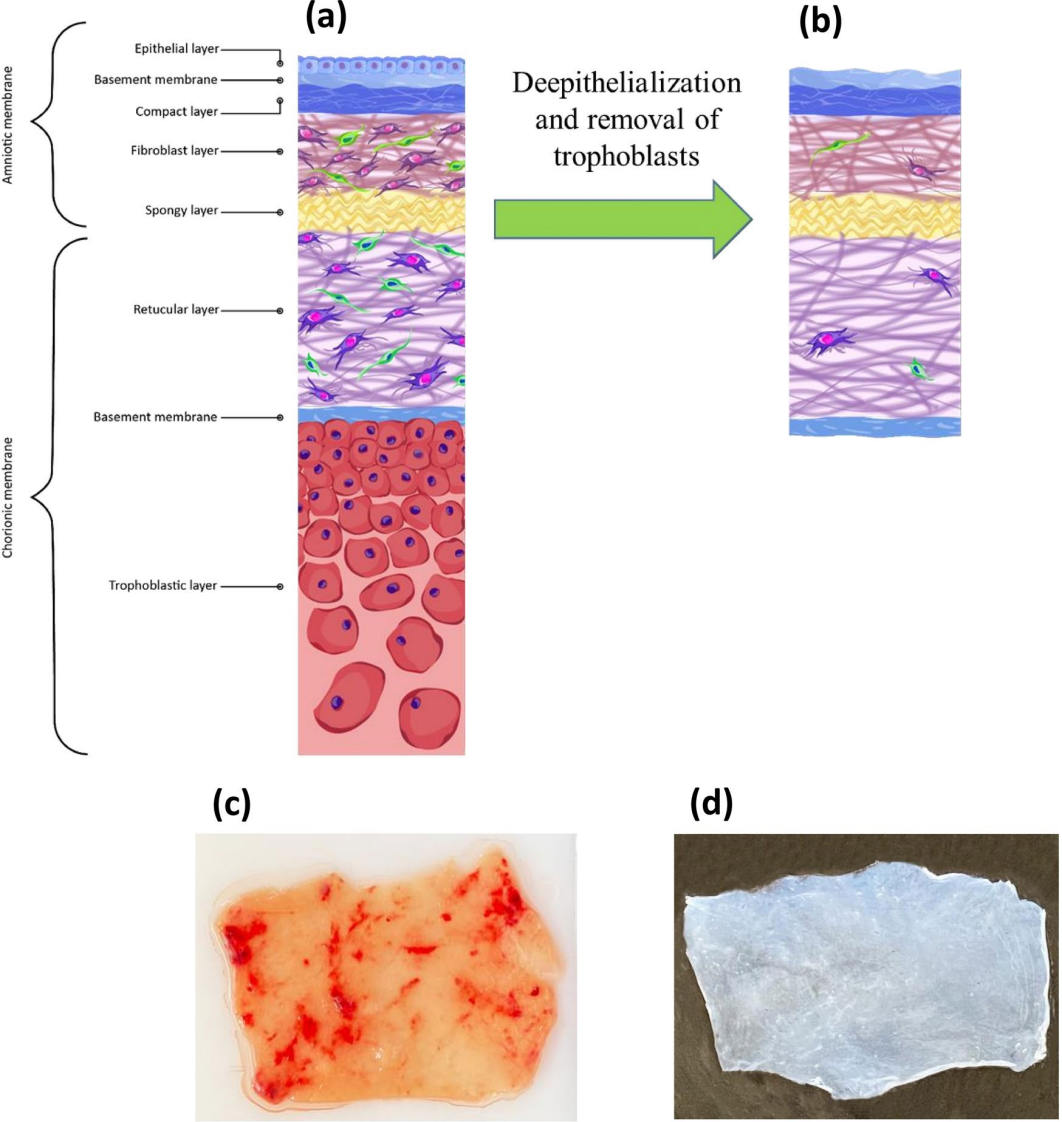
(**a**) Histological features of fresh amniochorionic membrane (fACM); (**b**) histological features of dbACM; (**c**) macroscopic features of fACM; macroscopic features of dbACM.

Placental membranes are one of the oldest biomaterials and allografts widely used in tissue engineering and regenerative medicine. Amniotic membrane and chorionic membrane are well-recognized natural scaffolds with exceptional properties^1^. The chorionic membrane contains more pro-angiogenic cytokines than a single amniotic membrane layer, which results in better tissue regeneration; while the amniotic membrane has more desirable biomechanical properties than the chorionic membrane^2–5^. Because amniochorionic scaffolds enjoy combined features of amnion and chorion, developing a scaffold with both of these layers has been the focus of many recent studies and clinical trials on reconstructive fields, including dermatology, orthopedic, ophthalmology, dentistry, and urology^6–8^. The amniochorionic membrane (ACM) exhibits noteworthy anti-inflammatory and anti-microbial properties. Furthermore, growth factors and cytokines of ACM promote angiogenesis and proliferation of endothelial and stem cells^9–12^. The amniotic membrane consists of a layer of epithelial cells, an underlying basement membrane, a compact layer, a fibroblastic layer and its mesenchymal cells, and a loose spongy layer that connects amnion and chorion, while the chorionic membrane consists of a reticular layer with mesenchymal cells, a basement membrane and the trophoblastic layer (Fig. 1a,b)^13^. The structural features of the basement membranes of amniotic and chorionic membranes made ACM a suitable substrate with desired mechanical properties that can provide two surfaces for cultivating a wide variety of cells^14^. Various cells, including cardiomyocytes, mesenchymal stem cells, fibroblasts, and limbal stromal cells, have been successfully cultured on placental membrane substrates^15–18^. These exceptional ACM properties can be attributed to the components of its extracellular matrix (ECM) and basement membranes. The basement membrane under the epithelial layer of amnion and the trophoblastic layer of chorion contains collagen type III, type IV and type V, laminin, and fibronectin which can act as a suitable bed for vascular growth^19^.

Biomaterials based on decellularized placental membranes such as decellularized amniotic membrane (dAM) or chorionic membrane (dCM) are increasingly attracting interest from many researchers^20,21^. Basement membranes under the epithelial layer of various tissues such as the aorta and gastrointestinal tract can act as an ideal substrate for cultivating a variety of cells since they provide the natural environment for cells^22–24^. However, decellularizing basement membrane without damaging the underlying components is necessary for recellularization and the ability of the substrate to support proliferation and migration of cells^25^. De-epithelialization of the amniotic membrane and removing the trophoblastic layer of chorion, to expose basement membranes reduces chances of graft rejection while improving the cell attachment and proliferation and increasing the product's shelf life. De-epithelialization of ACM can be achieved by employing physical and mechanical approaches such as freeze-thawing and scraping, which removes the epithelial layer, or chemical and enzymatic agents. Enzymatic agents such as Dispase, Trypsin, Thermolysin, DNAase, and chemical solvents such as Sodium dodecyl sulfate (SDS), NaOH, Triton X-100, Urea, and Thiourea loosen the connection between cells, lyse the cells, and remove antigens and DNA residues^9,26^. However, the majority of the decellularization methods focused on solely the decellularizing amniotic membrane or chorionic membrane. Therefore, de-epithelialization and removing the trophoblasts without separation of amniotic and chorionic membranes and any damage to the spongy layer (which contains various growth factors) is a new field of research. In many methods, these layers needed to be laminated together after the procedure. In other words, although decellularized basement membranes of the amniotic and chorionic membrane can be excellent culture grounds for stem cells, removing epithelial cells and trophoblastic layer without imposing considerable damage to the intermediate extracellular matrix during the process is still a challenging task.

Developing a natural bi-sided substrate could have potential application in corneal reconstruction (by culturing corneal endothelium and epithelium on each surface), skin reconstruction (by culturing keratinocytes and fibroblasts on each surface), vascular reconstruction (by culturing endothelial cells and vascular smooth muscle cells on each side), and cardiac reconstruction (by culturing endothelium and cardiomyocytes on each surface). Herein, with the aim of developing a bi-sided substrate with proper biomechanical properties of placental membranes, we developed a simple and reproducible protocol for de-epithelializing amniotic membrane and removing trophoblasts without damaging to the native basement membranes or destroying the middle spongy layer. The final product of this method is a de-epithelialized bi-sided amniochorionic membrane (dbACM), without trophoblastic layer and with an exposed basement membrane on each side, which has capability of supporting endothelial cell proliferation on two surfaces.

## Results

### Macroscopic properties and histological features

The de-epithelialization of the amnion and removal of trophoblasts resulted in a transparent scaffold without any visible clot, which could be easily handled without tearing. The scaffold’s texture and color were similar to the fresh amniotic membrane, but it was easier to handle and less likely to fold on itself during handling (Fig. 1c,d). Figure 1a,b demonstrates histological features of our scaffold compared to the fresh amniochorionic membrane (fACM).

**Figure 2:**
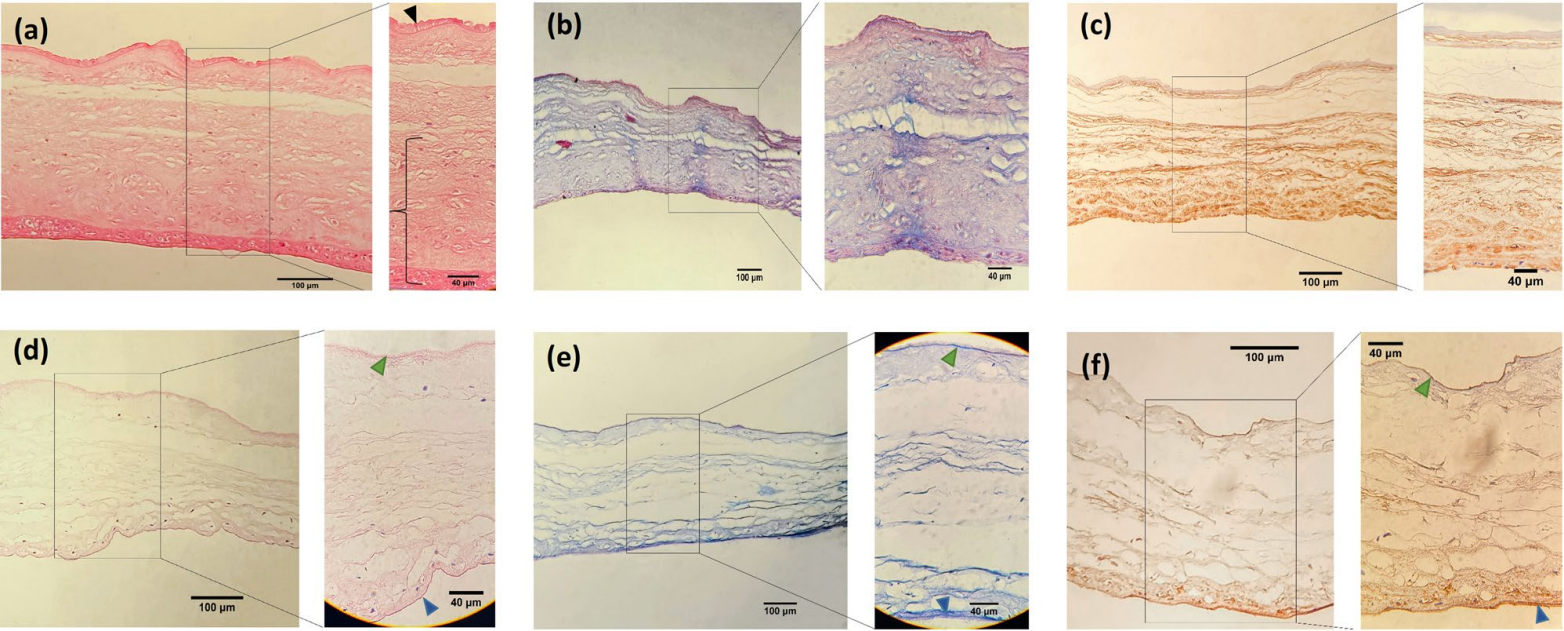
Histological features of fACM and dbACM; (**a**) H&E staining and (**b**) Mason’s trichrome staining of fACM; (**c**) IHC staining for collagen type IV in fACM; (**d**) H&E staining dbACM; (**e**) Mason’s trichrome staining of dbACM; (**f**) IHC staining for collagen type IV in dbACM; (black arrow: epithelial cells, black brace: trophoblasts, green arrow: amniotic basement membrane, blue arrow: chorionic basement membrane).

Hematoxylin and Eosin staining (H&E)﻿ staining was used for investigating the success of removing cells and morphology of the basement membranes of amniotic and chorionic sides. As demonstrated in Fig. 2a,d, it was observed that the epithelial layer of the amniotic membrane was removed, and the intact underlying basement membrane was exposed. Also, the trophoblastic layer of the chorionic membrane was completely removed while the underlying basement membrane was still intact. Although the most of the basement membranes of the chorionic and amniotic membrane remained in good condition, minor damages were seen, which was more prominent on the amniotic side. The spongy layer between two basement membranes was intact, as shown in Fig. 2d. Mason’s trichrome staining was used for evaluating the remaining collagen and basement membrane condition (Fig. 2b,e).The amnion and chorion’s basement membrane can be observed as the dark blue lines in Mason’s trichrome staining (Fig. 2e). Furthermore, to prove the integrity and existence of the basement membrane, we used immunohistochemistry staining for collagen type IV (Fig. 2c,f). As Fig. 2f demonstrates, the IHC staining for collagen type IV confirms the existence of the basement membranes on both amniotic and chorionic sides (dark brown lines).

### Biomechanical characteristics

The results and comparison between dbACM, fresh amniochorionic membrane, and cross-linked dbACM are demonstrated in Table 1. After de-epithelialization and removal of the trophoblastic layer, the scaffold was significantly thinner than the fACM. It was observed that decellularization slightly decreases the maximum load value, and the dbACM maximum load value remains at 6.04 ± 0.78 N. Furthermore, it was observed that Young’s module was also higher in dbACM. This observation can be attributed to the much lower thickness of the scaffold (311 µ vs. 184 µ). Furthermore, no significant difference was observed between dbACM and fACM in the suture retention test. Additionally, elongation at the point of the break was higher in fACM compared with dbACM. Another interesting observation during the biomechanical test was that at the maximum load of the scaffold and fACM, the chorionic layer was torn primarily. To assess the effect of cross-linking on biomechanical properties, we utilized glutaraldehyde. It was observed that cross-linking has no significant change in scaffold thickness. Furthermore, cross-linking by glutaraldehyde significantly increased the maximum load value and elongation at the point of the break. Cross-linking also improved suture retention test results compared with the scaffold. After cross-linking, the suture retention test and elongation at the point of the break were comparable to fACM.

**Table 1:** Biomechanical properties of dbACM, cross-linked dbACM, and fACM.

	fACM (F)	dbACM	Cross-linked dbACM	Significance (*p* value)		
				fACM vs dbACM	fACM vs Cross-linked dbACM	dbACM vs Cross-linked dbACM
Thickness [µ]	311.2 ± 38.52	184.0 ± 21.87	175 ± 21.28	*P* < 0.0001	*P* < 0.0001	*P* > 0.05
Max load F [N]	6.78 ± 0.73	6.04 ± 0.78	12.18 ± 0.70	*P* < 0.05	*P* < 0.0001	*P* < 0.0001
Young’s module [MPa]	0.73 ± 0.03	1.47 ± 0.17	2.33 ± 0.27	*P* < 0.001	*P* < 0.001	*P* < 0.0001
Suture retention [N]	0.69 ± 0.06	0.61 ± 0.08	0.78 ± 0.04	*P* > 0.05	*P* > 0.05	*P* < 0.005
Elongation [%]	47.68 ± 5.60	36.42 ± 3.26	42.54 ± 0.93	*P* < 0.001	*P* > 0.05	*P* < 0.05

### In vitro biodegradation test

**Figure 3:**
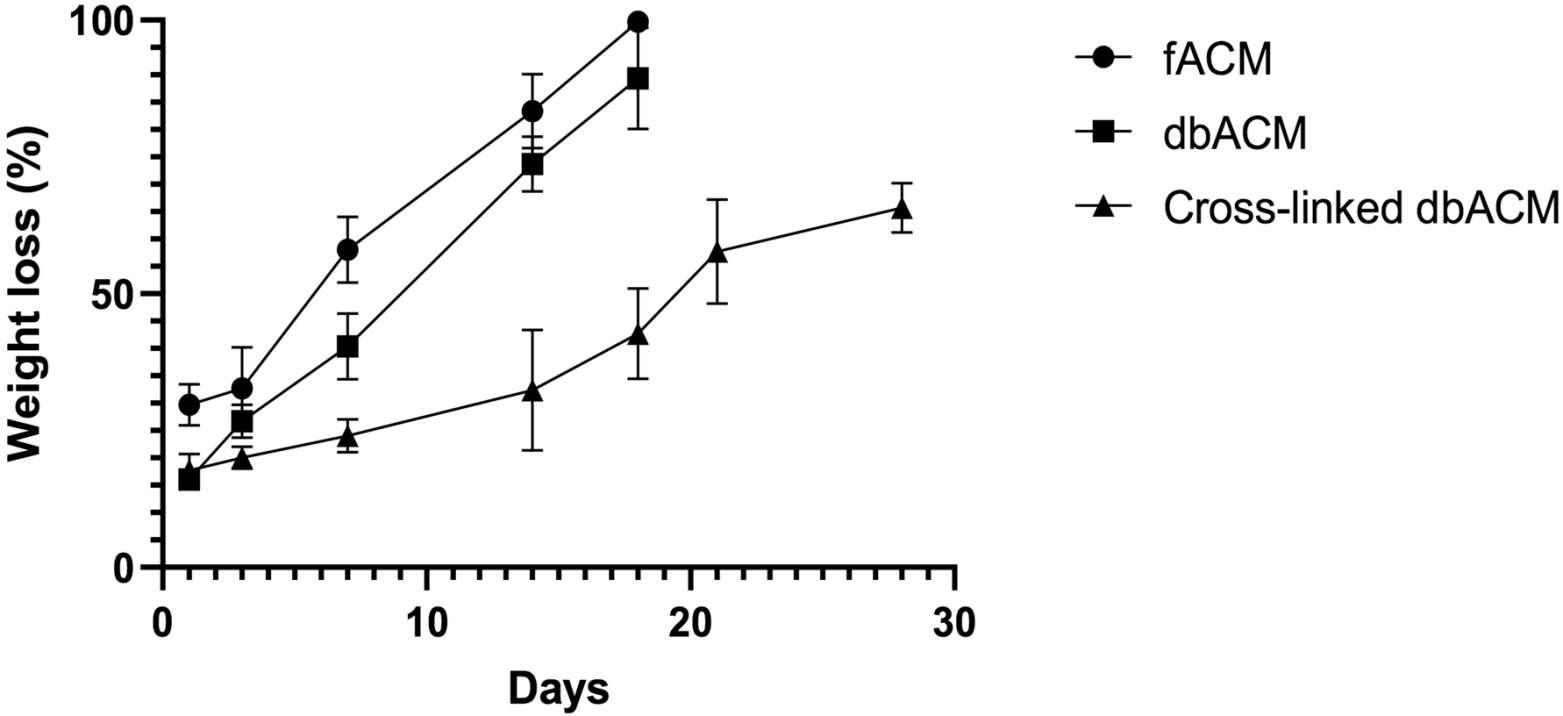
In vitro biodegradation test of fACM, dbACM, and cross-linked dbACM using crude collagenase with a concentration of 0.01%; values are expressed as the mean ± S.E.M (n = 3) results were analyzed using One-Way ANOVA followed by Tukey's post-Hoc test; analysis was conducted using GraphPad Prism version 9.

After one day in the biodegradation enzymes, dbACM and cross-linked dbACM lost near 15–20% of their weights. On day one, the weight loss was significantly higher (*p* value < 0.05) in fACM in comparison to dbACM and cross-linked dbACM, which can be attributed to the membranes' cellular components. Furthermore, it was observed on the 7th day of the experiment that the weight loss in fACM is much higher (*p* value < 0.05) than dbACM and cross-linked dbACM. By 18 days of incubation, nearly all of the fACM and dbACM scaffold were degraded by collagenase enzymes, and a jelly-like structure remained. However, the degradation rate in cross-linked specimens was significantly lower. It was observed by 28 days of incubation, only near 70% of the weight of the cross-linked scaffold was lost due to degradation (Fig. 3).

### Cytotoxicity, cell viability, and cell proliferation

**Figure 4:**
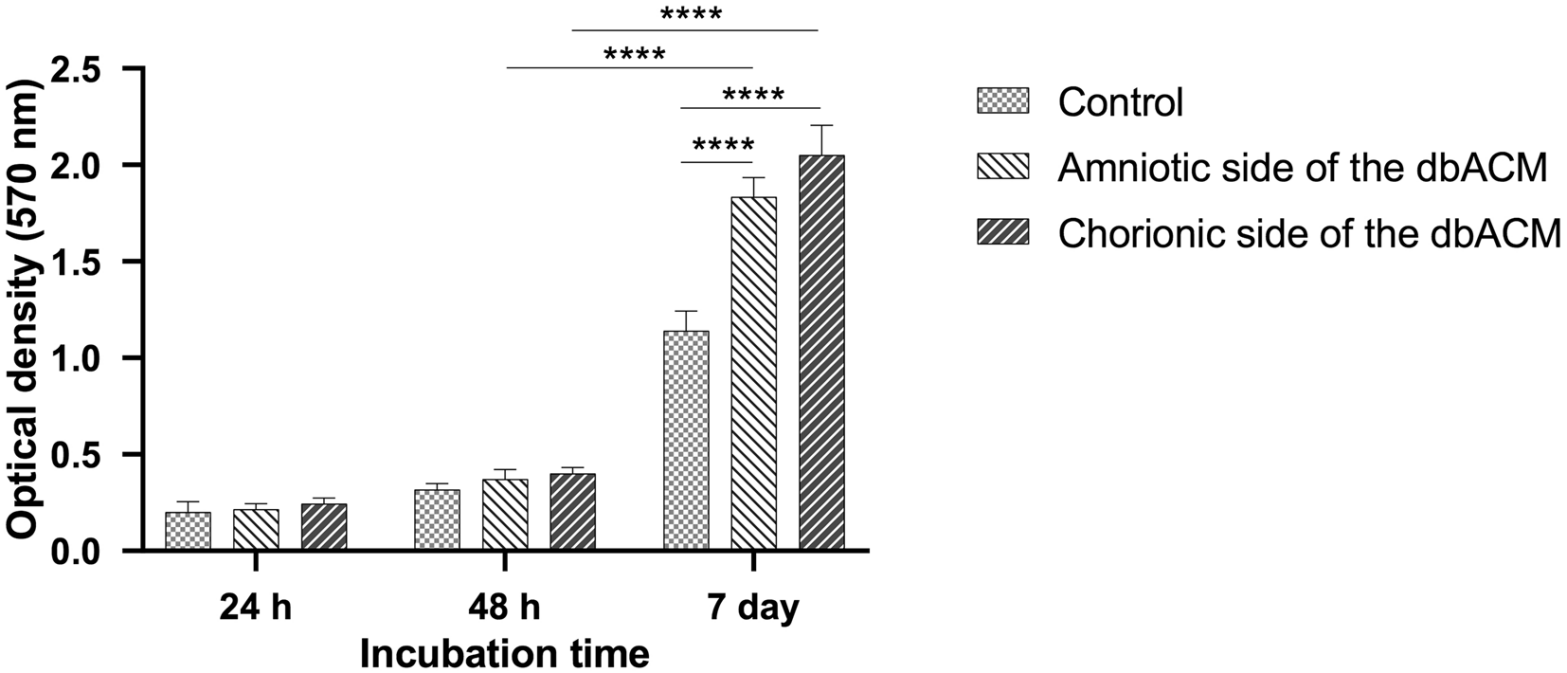
The results of MTT assays after culturing HUVECs on both amniotic and chorionic sides of dbACM and their comparison to the control group (HUVECs on culture plate); (****P < 0.0001, values are expressed as the mean ± S.E.M (n = 3) results were analyzed using One-Way ANOVA followed by Tukey's post-Hoc test); analysis was conducted using GraphPad Prism version 9.

The Human umbilical vein endothelial cells (HUVECs)﻿ were cultured on both the amniotic and chorionic sides of dbACM, and the cytotoxicity of both substrates and the cell viability was evaluated by MTT (3-(4, 5-dimethyl-2-thiazolyl)-2,5-diphenyl-2Htetrazolium bromide)﻿ assay after 24 h and 48 h of culture. Furthermore, we evaluated the proliferation of HUVECs and both surfaces by seven days of culture. The data were normalized to positive control that represented 100% cell viability. The results showed that the substrate is not cytotoxic. After 24 h of culture, there was no significant difference between the control group, the amniotic side of dbACM, and the chorionic side of dbACM. After 48 h of culture also no significant difference was observed between these three groups. After seven days of proliferation, both test subjects have a significantly higher optical density (OD) at 570 nm than the control group. Furthermore, these results suggest that the proliferation rate of HUVECs on the chorionic basement membrane is slightly higher than the amniotic basement membrane, but it was not significant (Fig. 4). This slight difference can be attributed to the higher concentrations of growth factors in the chorionic side.

### Scanning electron microscopy

**Figure 5:**
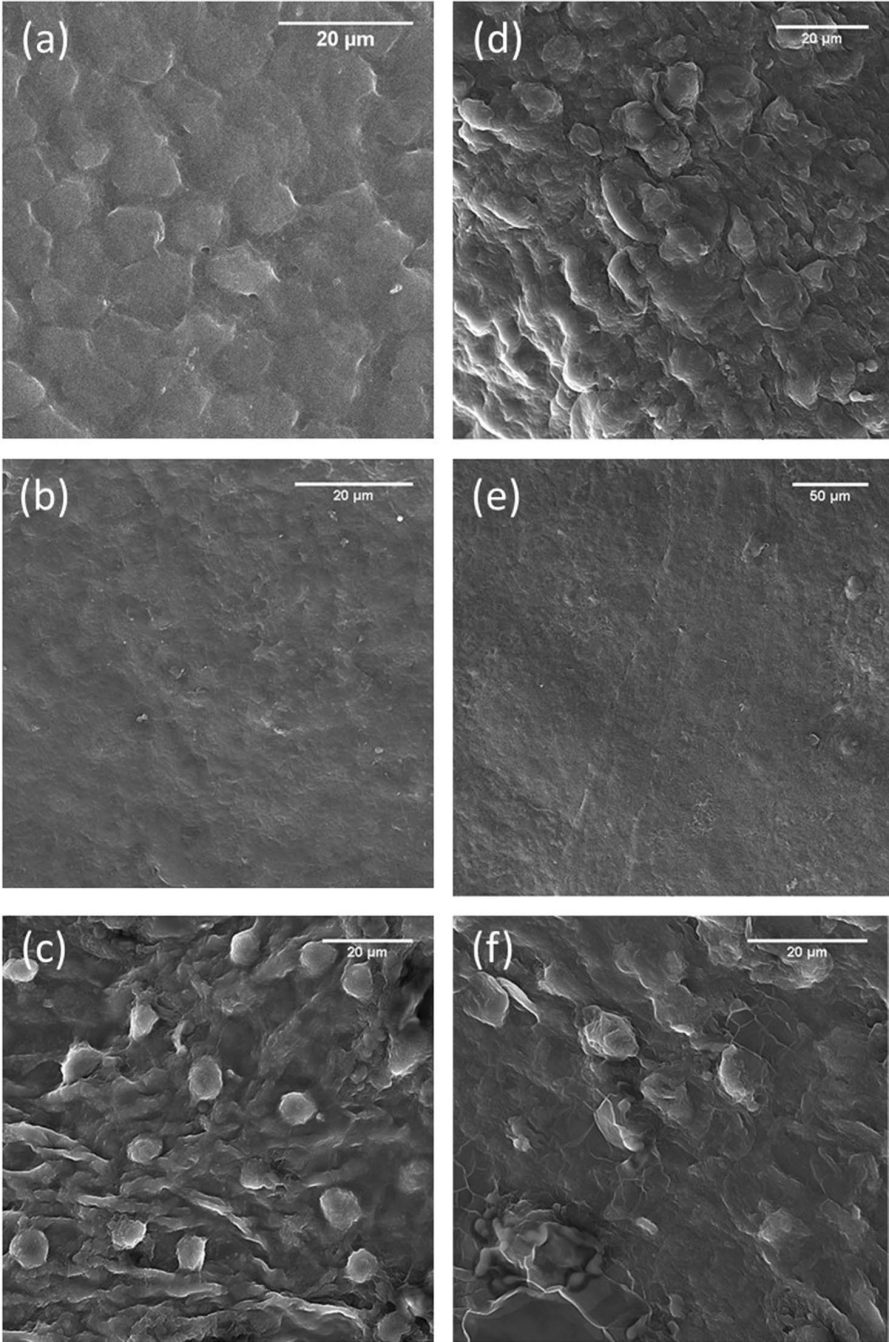
SEM images of dbACM before and after culturing HUVECs; (**a**) epithelial cells of amniotic membrane; (**b**) de-epithelialized amniotic membrane (basement membrane under the epithelial layer); (**c**) HUVECs on the amniotic side basement membrane of dbACM; (**d**) trophoblastic layer of chorion; (**e**) basement membrane under the trophoblastic layer after removing trophoblasts; (**f**) HUVECs on the chorionic side basement membrane of dbACM.

scanning electron microscope (SEM) was done for evaluating the results of decellularization protocol, basement membrane fibers, endothelial cell adhesion, and their morphology on both sides of the dbACM substrates. Figure 5a,d demonstrate the SEM images of fresh amniochorionic membrane. It was observed that the decellularization process could remove epithelial cells of the amniotic side and also trophoblasts of the chorionic side. The exposed underlying basement membranes were intact (Fig. 5b,e). For analyzing endothelial cell adhesion, HUVECs were cultured on both substrates for 5 days. It was observed that both amniotic and chorionic sides’ basement membranes could support the adhesion, growth, and proliferation of endothelial cells. SEM images demonstrated that HUVECs were attached to the surface of both basement membranes (Fig. 5c,f). Another observation was that in case of damage to the basement membrane by scraping, endothelial cells did not attach to the injury site. Elongated sprouts and cell-to-cell contact were observable, but the formations were not organized to form endothelial network assembly. In another observation, it was found out that in areas where the basement membrane was damaged, the HUVECs did not attach.

### Immunohistochemistry

**Figure 6:**
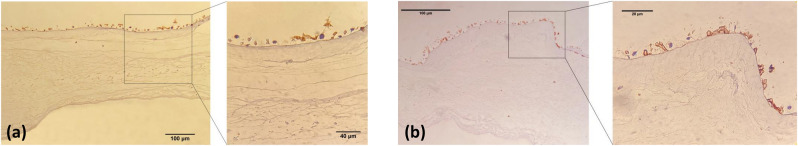
IHC for vWF after culturing HUVECs on both sides of dbACM; (**a**) amniotic side basement membrane of dbACM; (**b**) chorionic side basement membrane of dbACM.

To investigate endothelial cell adhesion and the effect of substrate on the characteristics of endothelial cells, the IHC analysis was conducted. As can be observed in Fig. 5, after 7 days of culture, von Willebrand factor (vWF) positive HUVECs were present on both surfaces of dbACM, indicating that HUVECs conserved their functional endothelial features (Fig. 6a,b).

### Endothelial adhesion molecule expression

**Figure 7:**
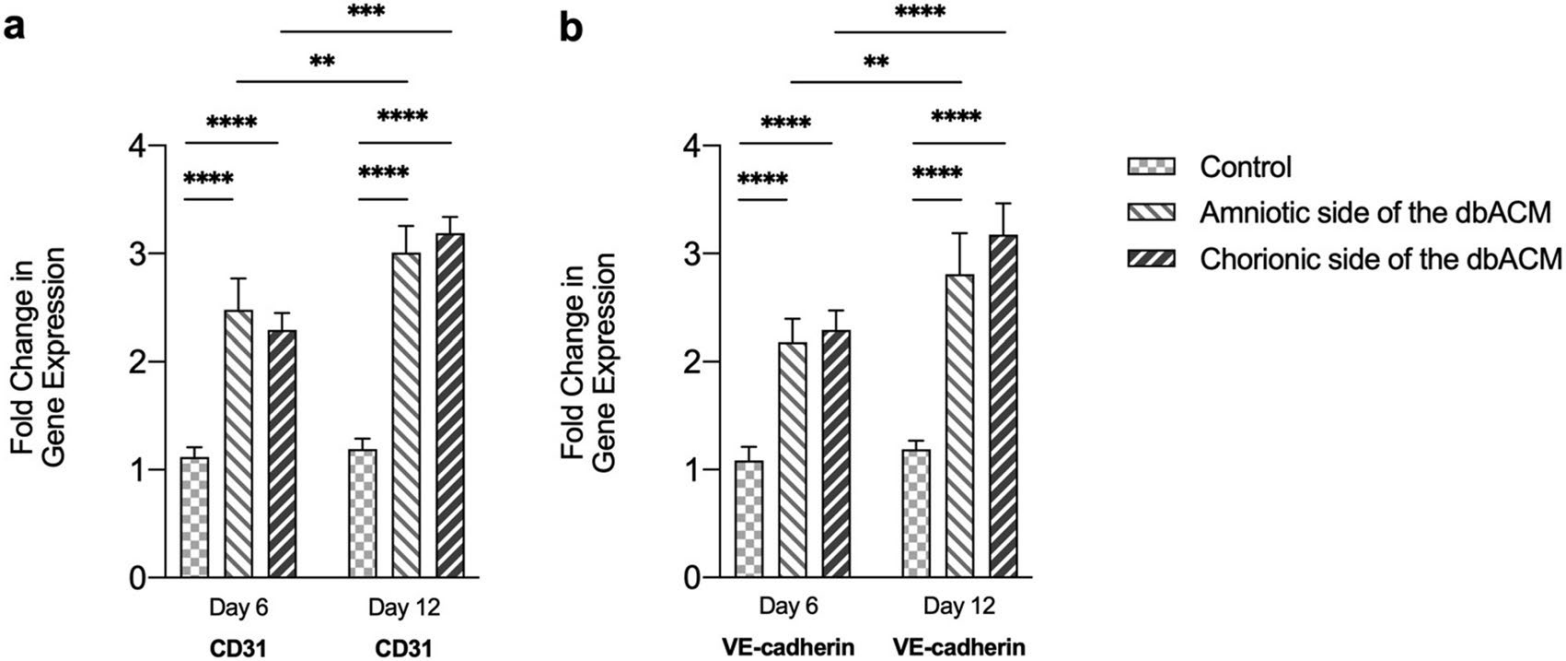
Evaluation of gene expression of CD31 and VE-cadherin in HUVECs cultured on amniotic and chorionic side of dbACM in days 6th and 12th. Values are expressed as the mean ± S.E.M (n = 5) results were analyzed using One-Way ANOVA followed by Tukey's post-Hoc test; Analysis was conducted using GraphPad Prism version 9.

In this step, the gene expression of VE-cadherin and CD31 in HUVECs was evaluated after culturing on two substrates. The results obtained from One-Way ANOVA analysis showed that the expression of both VE-cadherin and CD31 has been significantly increased after 6 and 12 days in HUVECs cultured on the amniotic and chorionic side of dbACM in comparison to the corresponding control group (Fig. 7). Furthermore, we observed higher expression of VE-cadherin and CD31 on the 12th day in comparison to 6th day in HUVEC cells cultured on amniotic and chorionic side of dbACM (*P* < 0.01 and *P* < 0.001, respectively).

### In vivo angiogenesis assay

**Figure 8:**
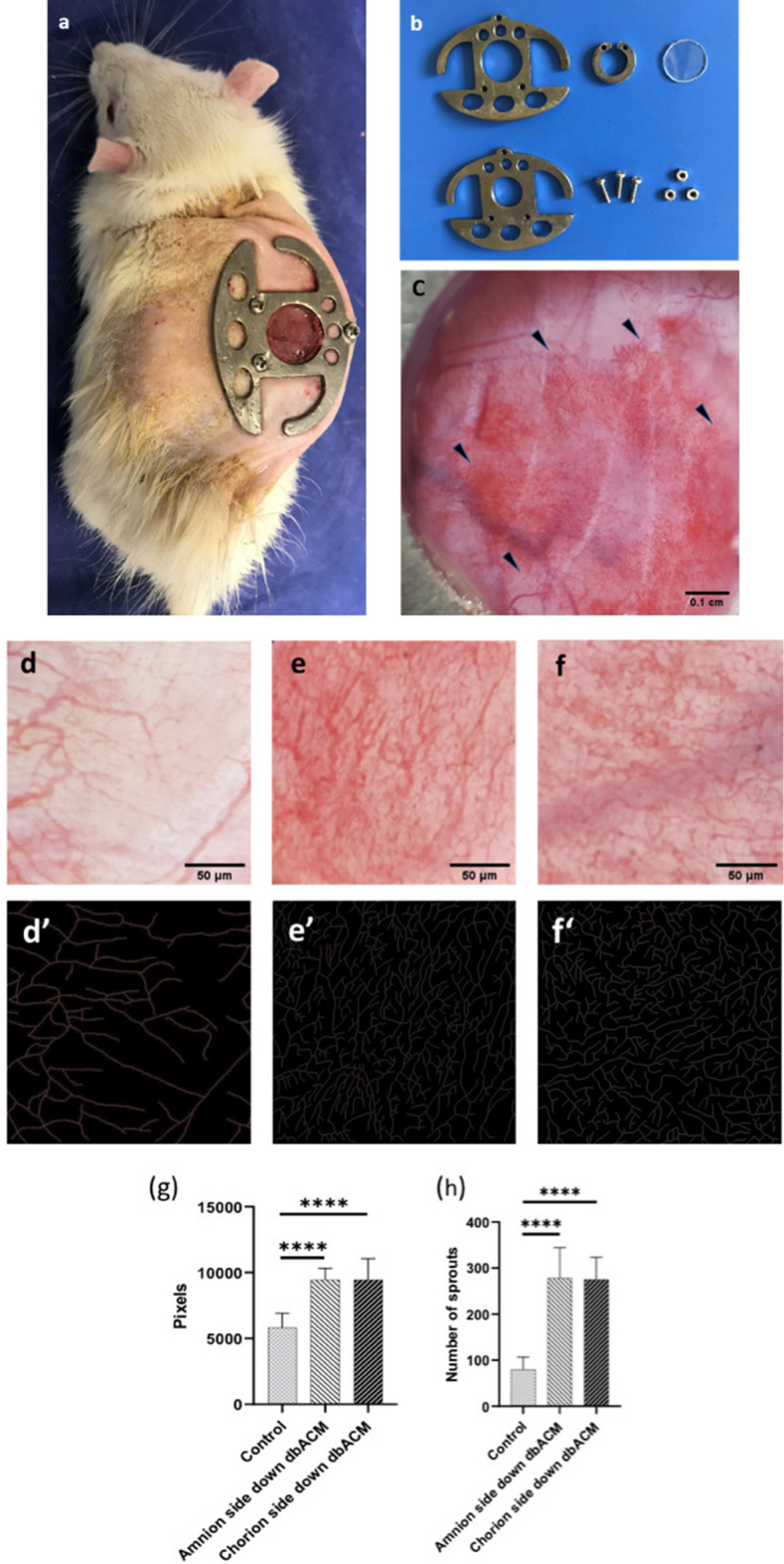
Result of dorsal skinfold chamber after 10 days of culture; (**a**) dorsal skinfold chamber mounted on rats; (**b**) dorsal skinfold chamber equipment; (**c**) skinfold chamber window after 10 days (angiogenesis induction is shown by the borders of dbACM implantation site with arrows); (**d**) control group (bared skin); (**e**) amniotic side down dbACM; (**f**) chorionic side down dbACM; (**d’**,**e’**,**f’**) ImageJ skeletonize picture of the same image as control, amniotic side down dbACM, and chorionic side down dbACM, respectively; result of ImageJ analysis: (**g**) total length of vessels in regions of interest; (**h**) number of branches in regions of interest; ****significance (*p* value < 0.001); values are expressed as the mean ± S.E.M; analysis was conducted using GraphPad Prism version 9; images were analyzed using ImageJ software with the Fiji plugin package (v.1.53c), which is based on ImageJ2 core.

From eight male rats, only one had signs of infection in the implantation site, which was excluded from the research. Results of the dorsal skinfold chamber model (n = 7) indicated that the both amniotic side and chorionic side of the dbACM can induce angiogenesis. ImageJ analysis indicated that after 10 days of culture, total vascular length and number of branches were significantly higher in amniochorionic substrate groups compared with the control group. Furthermore, no significant difference was observed between the chorionic side and amniotic side substrate regarding their pro-angiogenic capabilities. These results are summarized in Fig. 8.

## Discussion

Placental membranes can be manipulated to achieve different goals such as easy handling, enhanced angiogenic abilities, conserved biomechanical properties, and improved shelf-life. Herein, aiming to develop a scaffold with two substrates for culturing the cells, we developed a method for removing the epithelial layer of the amnion and the trophoblastic layer of the chorion. The final product is a bi-sided substrate with the capability of supporting endothelial cell proliferation on two surfaces, which can be used as a graft with various clinical applications. This method optimizes mechanical and enzymatic decellularization processes using Trypsin 0.1% w/v, ethylenediaminetetraacetic acid (EDTA) 0.1% w/v, and streptokinase 0.02% w/v with gentle scraping. In some methods, the amniotic and chorionic membrane will be separated at the start and laminated after decellularization and dehydration to develop grafts with different applications in wound healing and surgical repair^27–30^. However, there are many efforts to conserve the most components of amniochorionic layers, especially the spongy layer; in some of them even with an increased risk of graft rejection due to remaining clots trapped in this layer. The loose jelly-like spongy layer, which contains a wide variety of anti-inflammatory and angiogenic growth factors, can be easily separated in the scaffold preparation process during the dissociation of amniotic and chorionic membranes^31,32^.

Furthermore, herein we decided not to manipulate the mesenchymal cells of the inner layers of the amniochorionic membrane. These cells exist in the amnion’s fibroblast layer and chorionic reticular layer, and the isolation of these cells requires total enzymatic digestion of the membrane by collagenase enzymes^33^. It has been observed that both amniotic mesenchymal cells and chorionic mesenchymal cells have an immunomodulatory effect and can prevent graft rejection and inflammatory response^34–36^. Furthermore, it has been suggested that the mesenchymal cells of the amnion have pro-angiogenic abilities and can promote angiogenesis after implantation^37–39^. Moreover, amniotic mesenchymal cells contain various angiogenic growth factors, including angiopoietin, vascular endothelial growth factor (VEGF), and basic fibroblast growth factor (bFGF)^40,41^. With that in mind, many researchers tried to preserve the mesenchymal cells in the final scaffold^4^. In our previous study, we have observed that mesenchymal cells of the amniochorionic membrane have pro-angiogenic properties, while the existence of epithelial layers can prevent angiogenesis^42^. Based on the results of our previous study, we removed the epithelial layer while maintaining the amniochorionic layers other than the trophoblastic layer to enhance the pro-angiogenic abilities. Furthermore, it has been demonstrated in clinical trials previously that these cells can be conserved in the grafts and the scaffold without the fear of adverse reactions^29,31,32,43–45^.

We decided to remove the trophoblastic and epithelial layers of human ACM with minimal damage to the underlying basement membranes to reduce the risk of rejection while conserving the spongy layer, biomechanical properties, inner layer cells, and easy handling of the scaffold. One of the aims of this study was to remove clots that were originated from the placenta and trapped in the spongy layer without disturbing this layer. Unlike the decellularization of the amniotic membrane in which most of the clots sticking to the spongy layer which can be removed during the washing process, removing clots from the amniochorionic membrane without disturbing or separating these layers is a challenging task. Furthermore, the chorionic membrane is not avascular, and the micro-clot still exists in the chorion's microvessels these clots are hard to remove during the washing process and need to be removed by streptokinase.

Streptokinase was used to dissolve trapped microscopic clots in the trophoblastic and spongy layer of the amniochorionic membrane. Streptokinase is a thrombolytic enzyme that is used in the treatment of patients with pulmonary thromboembolism and myocardial infarctions^46^. Additionally, this fibrinolytic enzyme can also unbind cellular adhesions and help to decellularize the natural substrate^47^. In the future studies, we aim to improve the decellularization method by trying alternative combinations with streptokinase. For instance, decellularization using urea-based solvents has been used with promising results in decellularizing and homogenizing natural scaffolds like adipose tissues^48,49^. Furthermore, urea has been utilized for de-epithelizlizing amniotic membrane^50,51^. This agent can also be used for neutralizing streptokinase and inhibiting unwanted damages to the basement membranes. However, using urea requires further trial and error since it can trigger denaturation of other proteins than streptokinase in high concentrations^52^. The final product of our method was a transparent scaffold without any visible or microscopic clots. The presence of clot could result in inflammation and possible graft rejection.

H&E staining showed that the process removed the epithelial layer and trophoblastic layer. Furthermore, Mason’s trichrome staining, SEM images, and IHC for collagen type IV showed that the basement membrane underneath these two layers remained intact. Removing these layers exposes the even basement membranes, which have an extensive ability for supporting various cells like mesenchymal stem cells of different origins or fibroblasts and endothelial cells^53–55^.

The maximum load value for fresh amnion membrane has been reported about 3–4 N, and 2–3.5 N for the chorionic membrane, which both are significantly decreased after the majority of decellularization procedures. Biomechanical measurements indicated that compared to placental grafts with similar applications, a maximum load value of 6.04 ± 0.78 N is totally acceptable for dbACM scaffold^53,55–57^. We observed in the stress–strain test that the chorionic side of our scaffold was torn first. This finding was not unexpected since the most of the scaffold's strength can be attributed to the amniotic layer rather than the chorionic layer. The suture retention test was an essential part of our investigation since one of the aims of the future of this study is to evaluate the scaffold's potential ability to be used as a surgical graft. Although the placental membranes have been used as surgical grafts, their suturability is one of their limitation compared to the other biomaterials which leads to developing alternative strategies such as using adhesive biocompatible hydrogels for attaching amnion membrane^58^. The suture retention test showed that this scaffold is completely suturable and can be used as a surgical graft. Suture retention near 0.6 N is enough for application in ophthalmic and microvascular surgeries^56,59,60^. Furthermore, to evaluate the cross-linking process on biomechanical properties of dbACM, we used glutaraldehyde with a concentration of 0.01% as a cross-linking agent. It was observed that cross-linking improved scaffold's resistance to collagenase in in vitro biodegradation tests. Furthermore, the cross-linking significantly improved the handling of the dbACM. Glutaraldehyde has been used with different concentrations for cross-linking of amniotic membrane for many years^61^. Previous studies stated that the cross-linking by glutaraldehyde preserves amniotic basement membrane ability for cellular growth. Although we used rather a low concentration of glutaraldehyde in this study (0.01), higher concentrations of glutaraldehyde have been used with little concerns about cytotoxicity^62,63^. In addition to glutaraldehyde, the other agents and methods can be used for cross-linking, which should be evaluated in the future studies^64,65^.

Since one of the primary objectives of this study was developing a scaffold with pro-angiogenic characteristics and the ability to support endothelial cell proliferation, we selected HUVEC as representative of endothelial lineage cells. After one week of culturing HUVECs on both substrates, an expanded monolayer of the cells was observed. The SEM images of the samples after 5 days of culture demonstrated the cell adhesion. vWF, a glycoprotein in endothelial cells which plays an essential role in hemostasis, is often used to detect endothelial cell characteristics^66,67^. The results of immunohistochemistry staining against the vWF showed that the amniotic and chorionic sides of the scaffold can be an appropriate bed for endothelial growth without altering the endothelial characteristic. Furthermore, it was concluded from the results of the MTT assay that there was no significant difference between the proliferation rates of endothelial cells on the basement membranes of both substrates. Moreover, evaluation of the endothelial adhesion molecules’ expression (vascular endothelial cadherin (VE-cadherin) and CD31) confirmed that both of the basement membranes are capable of supporting the proliferation of endothelial cells. VE-cadherin (or CD144 and CD31 have been frequently used for the evaluation of endothelial cell activity, adhesion, and proliferation^68,69^. The dorsal skinfold chamber model is an established form of intravital microscopy that has been frequently used as an angiogenesis model to evaluate the angiogenic properties of different scaffolds. Several methods have been used for analyzing dorsal skinfold chamber images for assessing the effect of scaffolds on promoting angiogenesis. We analyzed the number of branches and the total length of branches in each region of interest by analyzing skeletonize images of the region of interest after turning the picture into the binary image as previously performed^70^. Just like the amniotic membrane, the dbACM could adhere to the dorsal skinfold window without any help from adhesive materials or sutures. After implantation, it was observed that both the chorionic and amniotic side of the dbACM promoted angiogenesis compared to the control group, and there was no significant difference between the chorionic and amniotic substrates regarding its angiogenic characteristics. These results from in vivo implantation are in line with our previous results from MTT assays which indicated that there was no significant difference between the chorionic side and amniotic side of dbACM regarding their angiogenic abilities. Our observations can be attributed to the pro-angiogenic growth factors such as VEGF in the middle layers of the amniochorionic membrane. Additionally, conserving the spongy layer with the current decellularization method can also contribute to the pro-angiogenic properties of dbACM.

## Conclusion

In order to develop a scaffold with two surfaces for cell culture, we decellularized amniochorionic membrane without the separation of two layers and removed the epithelial layer of amnion and the trophoblastic layer of the chorion. Tissues like the vessels, cornea, gastrointestinal tract, and urethral tract have a stroma with unique biomechanical properties sandwiched between two expanded layers of cells. For instance, endothelial cells form the lining of the luminal side of the vessels, while the abluminal side consists of smooth muscle cells. In another example, in the cornea, the stroma is between two expanded layers of corneal endothelium and epithelium. Additionally, cultivating endothelial cells on each side would improve the pro-angiogenic properties of the scaffolds and substrates. Therefore, several experiments have been conducted to achieve a scaffold with two substrates for cultivating various cells. The decellularized amniochorionic membrane has an exposed basement membrane on each side, which could be used for cultivating a wide variety of cells. Herein, we demonstrated the pro-angiogenic capability of the substrate and its ability to support endothelial cell proliferation on both surfaces. These results suggest that a scaffold with two basement membranes as substrates could possibly support the proliferation of osteoblasts, limbal stromal cells, smooth muscle cells, and cardiomyocytes. In addition, the bi-sided amniochorionic scaffold would be a promising biomaterial to be used in skin flaps, cardiac patches, vascularized composite allografts, and microvascular tissue engineering. Future studies are required to translate the results of our study into clinical applications.

## Materials and methods

### Obtaining placenta tissue and preparing amniochorionic membrane

Placenta tissues (n = 10) were collected from an elective cesarean section with the parents’ written informed consent. All of the procedures in this study were performed in accordance with the declaration of Helsinki and under the supervision and approval of Shahid Beheshti University of Medical Sciences (SBMU) Ethics committee and according to SBMU policies on medical and research ethics (Code: IR.SBMU.MSP.REC.1399.466). All mothers who contributed to this study were tested negative for human hepatitis virus types B and C, human immunodeficiency virus types 1 and 2, cytomegalovirus, syphilis, gonorrhea, and toxoplasmosis. Moreover, there were no signs of premature rupture of the membrane or history of prenatal infection. The reason for selecting placentas from elective cesarean sections and excluding conditions that can cause oxidative stress in placentas was to limit the number of membrane micro-fracture and remove conditions that can cause histological disturbances in the membrane^71,72^.

All mothers were between 39 and 41 weeks of pregnancy, and pre-term or post-term deliveries were excluded. After delivery, the placental tissues were maintained in normal saline serum at 4–8 °C under sterile condition and immediately transferred to the laboratory. All the procedures were performed in a class 2 laminar flow under sterile conditions, within an hour after surgery. A 10 × 15 cm segment of the amniochorionic membrane was dissected from the placenta with at least 3 cm margin from the placental disc and washed several times in phosphate buffer saline (PBS) (pH 7.4) to remove visible blood clots. Furthermore, after macroscopic observation of all specimens for signs of abnormality, we used H&E staining to evaluate the histological properties of amniochorionic membranes to rule out tissues with chorioamnionitis, laminar necrosis, signs of infection, and premature rupture of membrane. All specimens of this study were negative for these abnormalities.

### Decellularization of amniochorionic membrane

The ACM was flattened in Trypsin-EDTA diluted with PBS (Trypsin–EDTA, 0.1% w/v, Sigma-Aldrich, USA) for 20–30 min at 37 °C. After washing the membrane in Dulbecco’s modified Eagle’s medium (DMEM) culture medium (pH 7.4), the membrane was incubated in streptokinase (0.02% w/v, Sigma-Aldrich, USA) for 8–10 min at 37 °C and subsequently, the membrane was washed with sterile DMEM culture medium (pH 7.4) once and lastly with PBS three times.

Following these treats, trophoblastic cells of the chorionic side of the membrane were gently scraped out in PBS by a plastic scraper without detaching the amniotic membrane from the chorionic membrane or rupture of the membrane for 10–15 min at 25 °C. Epithelial cells of the amniotic side of the membrane were scraped out gently by scraping with the same strength in PBS for 10–15 min. Finally, the scaffold was washed in PBS three times.

### Cross-linking of decellularized amniochorionic membrane

In this study, we selected glutaraldehyde which has been frequently used for cross-linking of amniotic membrane^73^. After decellularizing the amniochorionic membrane, the scaffold was cross-linked using 0.1% glutaraldehyde (10 mM) for 30 min at room temperature.

### Biomechanical analysis

The biomechanical properties of dbACM were evaluated and also compared with the natural fresh amniochorionic membrane and cross-linked scaffold (cross-linked dbACM). The biomechanical analysis was conducted in Polymer and Petrochemical Institute (IPPI). The average thickness of the samples was measured by a caliper (Absolute AOS Digimatic Caliper, Mitutoyo Europe GmbH, Germany). The maximum load value, maximum elongation on the breaking point, and suture retention strength were measured using a uniaxial universal test machine (STM-20, Santam), with an elongation speed of 10 mm min^−1^ with a samples size of 20 × 40 mm. For evaluating the suture retention strength, one side of the samples was sutured (10 mm from the edge) with nylon 5–0 round suture, while the opposite edge of the samples was tightly held in clamps of the testing machine. Since the thickness of the spongy layer can significantly decrease after dehydration, which would interfere with biomechanical results during the biomechanical analysis, we used PBS to hydrate the samples.

### In vitro biodegradation

The biodegradation properties of the scaffold were investigated and compared with the natural fACM and cross-linked scaffold, using in vitro enzymatic digestion. The samples were cut into 10 × 10 mm pieces and were immersed in 1 cc of the biodegradation solution of collagenase H (Roche, Germany) diluted with PBS (0.01% w/v) (pH 7.4) in a 24-well plate and stored at 37 °C. The samples were followed on days 1, 3, 7, 14, 18, 21, and 28. On each day, samples were removed from the solution and subsequently weighed.

### Histological analysis

The scaffold was stored in 10% formalin for 24 h and fixed using DID SABZ Co. DS 2080/H tissue processor. H&E technique was used for evaluating histological properties of the scaffold. For further evaluation of ECM content, the scaffold was stained with Mason’s trichrome staining technique. Furthermore, to evaluate the integrity of the amniotic and chorionic basement membrane, IHC staining for the presence of collagen type IV was conducted. Collagen type IV has been frequently used for demonstrating the existence of basement membranes in various tissues including placental membranes^14,74^. Also, after the decellularization process, the chorionic side and amniotic side of the scaffold are quite similar in the H&E staining. The collagen type IV is highly expressed in both amniotic and chorionic basement membranes also expressed in the reticular layer of chorion but not in the compact layer and fibroblast layer of amnion^14^. This means the IHC for collagen type IV can also help differentiate the chorionic side and amniotic side of the scaffold.

### Cytotoxicity, cell viability, and cell proliferation

HUVEC line was purchased from Stem Cell Technology Research Center (STRC) and cultured in a medium suggested by the provider consisting of Dulbecco’s modified Eagle’s Medium/Ham’s Nutrient Mixture F-12 (DMEM/F12) + 10% FBS + 90 U/ml heparin and 1% penicillin–streptomycin solution. The HUVECs were cultured on both the chorionic side and amniotic side substrates in a 24-well plate with a density of 40,000 cells/well, in the mentioned media and incubated for 24 h, 48 h, and 7 days (d) under 95% air and 5% CO_2_ at 37 °C. HUVECs cultured on standard wells were used as the positive control. To investigate cytotoxicity, cell viability, and cell proliferation rate of the substrate, the MTT assay was used. Briefly, 2.5 cc of MTT solution was added to the plates and placed on an incubator for 3–4 h. The formed formazan crystals were dissolved by adding 1.5 ml of Dimethyl Sulfoxide (DMSO) (Sigma-Aldrich, USA) solution. The negative control was the wells with DMEM without scaffold and cells. The optical density (OD) of the solution at a wavelength of 570 nm was observed by a spectrophotometer (Cecil BioQuest CE 2501, UK). The blank OD was subtracted from the OD of the other groups. MTT assay was done in 24 h, 48 h, and 7 days of culture.

### Scanning electron microscope

SEM (TECAN-VEGA-II, Czech Republic) was used to investigate the amniotic and chorionic basement membrane after the decellularization process and also endothelial cell adhesion to the substrate. Tissue samples were prepared for SEM as previously described^75^. Briefly, dbACM scaffolds and cell-seeded dbACM scaffold were fixed using paraformaldehyde 10% solution for one hour and then dehydrated using an ethanol graded concentration of 30%, 50%, 70%, 80%, 90%, and twice of 100% for 10 min. After coating samples with gold by sputtering, SEM images were taken and analyzed at an acceleration voltage of 15 kV. For investigating the morphology of the cells adhered to the substrate, HUVECs were cultured with a density of 4 × 10^4^ per cm^2^ on both the chorionic side and amniotic side substrate in a growth media consists of DMEM/F12 + 10% FBS + 90 U/ml heparin and 1% penicillin–streptomycin and incubated in 95% air and 5% CO_2_ at 37 °C for five days. The culture media was changed every 48–72 h.

### Immunohistochemistry

For evaluating the capability of the amniochorionic membrane to act as a substrate for supporting cells and endothelial cell adhesion, the cell-seeded dbACM substrate were assayed by immunohistochemistry. For this purpose, HUVECs with a density of 4 × 10^4^ per well were seeded on both the chorionic side and amniotic side substrate in a 24-well plate with a growth media of DMEM/F12 + 10% FBS + 90 U/ml heparin and 1% penicillin–streptomycin and incubated in 95% air and 5% CO_2_ at 37 °C. The culture media was changed every 2–3 days. After five days of culture, the samples were fixed with paraformaldehyde 10% solution, and the scaffold was processed and stained with anti-von Willebrand factor for assessing endothelial characteristics.

### RT-qPCR assessment for evaluating the RNA and protein expression

To evaluate endothelial adhesion molecules changes, we used reverse transcription-polymerase chain reaction (RT-PCR) by using the corresponding primers, reagent kit (Takara Bio Inc., Otsu, Japan), and light cycler device (Roche Diagnostics, Mannheim, Germany). In this regard, total RNA was extracted using Trizol reagent from the samples, and Gene-level alterations in mRNA were calculated using RT-qPCR after the reverse transcription of 1 μg of RNA from each sample using the reagent kit. Primer sequences have been designed based on previous reports using SYBR Premix Ex Taq technology (Takara Bio Inc., Otsu, Japan) as follows: Forward primer of 5′-TTGGAACCAGATGCACATTGAT-3′ and reverse primer of 5′-TCTTGCGACTCACGCTTGAC-3′ for VE-cadherin and forward primer of 5′-AACAGTGTTGACATGAAGAGCC-3′ and reverse primer of 5′-TGTAAAACAGCACGTCATCCTT-3′ for CD31. Thermal cycling conditions included an initial activation step for 30 s at 95 °C afterward 45 cycles, as well as a denaturation step for 5 s at 95 °C and a combined annealing/extension step for 20 s at 60 °C. Analysis of the melting curve was conducted to certify if all primers yielded a single PCR product.

### In vivo implantation in dorsal skinfold chamber

For evaluating the pro-angiogenic capability of the substrates and the ability to support endothelial proliferation in vivo, we utilized the dorsal skinfold chamber model. All of the animal surgeries and in vivo procedures were approved and conducted under the supervision of SBMU ethics committee policies on animal research (Code: IR.SBMU.MSP.REC.1399.466) and also according to Animal Research: Reporting In Vivo Experiments (ARRIVE) guidelines. Dorsal skinfold chamber analysis was conducted as we described previously^42^. Briefly, 4–6-week-old male rats weighing between 180 and 200 g were selected and anesthetized using intraperitoneal injection of Ketamine with a dose of 80 mg/kg and Xylazine with a dose of 10 mg/kg. After sedation, the dorsal skin of the rats was shaved at the site of the surgery. Sterilized custom-made platinum chambers were mounted on the rats’ dorsal skinfold and stabilized. One side of the fold’s skin was removed in a circle with 1 cm diameter using a scalpel with blade No.15. After removing the skin, the dorsal skinfold window site was prepared and covered by a sterile glass to make a dorsal skinfold chamber. 24 h after surgery, both amniotic side-down and chorionic side-down dbACM with a size of 5 × 5 mm were implanted in the dorsal skinfold chamber and observed for 10 days. In each dorsal skinfold chamber, four regions with a size of 0.1 × 0.1 mm were randomly selected from the central part of the model where the dbACM was previously implanted. The final analysis was conducted using the open-source ImageJ software with the Fiji plugin package (v.1.53c), which is based on ImageJ2 core (imagej.net/software/fiji/). After the initial enhancement of images, the pictures turned into binary pictures, and after skeletonizing process, the Analyze Skeleton plugin was used for evaluating the number of branches and the total length of vessels in the selected regions.

### Statistical methods

All analyses were done using GraphPad Prism version 9. We used one-way ANOVA followed by Tukey’s post-Hoc test for statistical analysis. *P* value less than 0.05 was considered significant.

## Data Availability

The datasets used and/or analyzed in the study are available from the corresponding author upon reasonable request.
